# Can malaria rapid diagnostic tests by drug sellers under field conditions classify children 5 years old or less with or without *Plasmodium falciparum* malaria? Comparison with nested PCR analysis

**DOI:** 10.1186/s12936-018-2508-x

**Published:** 2018-10-16

**Authors:** Freddy Eric Kitutu, Henry Wamani, Katarina Ekholm Selling, Fred Ashaba Katabazi, Ronald Bisaso Kuteesa, Stefan Peterson, Joan Nakayaga Kalyango, Andreas Mårtensson

**Affiliations:** 1 Department of Women’s and Children’s Health, International Maternal and Child Health (IMCH), Uppsala University, Uppsala, Sweden; 2 Pharmacy Department, Makerere University College of Health Sciences, Kampala, Uganda; 3 School of Public Health, Makerere University College of Health Sciences, Kampala, Uganda; 4 UNICEF, Health Section, 3 UN Plaza, New York, NY 10017 USA; 5 Clinical Epidemiology and Biostatistics Unit, Makerere University College of Health Sciences, Kampala, Uganda; 6 Department of Medical Microbiology, Makerere University College of Health Sciences, Kampala, Uganda; 7 Infectious Disease Institute, Makerere University College of Health Sciences, Kampala, Uganda

**Keywords:** Integrated case management, Rapid diagnostic test, Polymerase chain reaction, Drug seller, Uganda, Febrile illness, Malaria, Private sector, Compliance, External quality assurance

## Abstract

**Background:**

Malaria rapid diagnostic tests (RDTs) available as dipsticks or strips, are simple to perform, easily interpretable and do not require electricity nor infrastructural investment. Correct interpretation of and compliance with the RDT results is a challenge to drug sellers. Thus, drug seller interpretation of RDT strips was compared with laboratory scientist re-reading, and PCR analysis of *Plasmodium* DNA extracted from RDT nitrocellulose strips and fast transient analysis (FTA) cards. Malaria RDT cassettes were also assessed as a potential source of *Plasmodium* DNA.

**Methods:**

A total of 212 children aged between 2 and 60 months, 199 of whom had complete records at two study drug shops in south western Uganda participated in the study. Duplicate 5 μL samples of capillary blood were picked from the 212 children, dispensed onto the sample well of the CareStart™ *Pf*-HRP2 RDT cassette and a FTA, Whatman™ 3MM filter paper in parallel. The RDT strip was interpreted by the drug seller within 15–20 min, visually re-read centrally by laboratory scientist and from it; *Plasmodium* DNA was recovered and detected by PCR, and compared with FTA recovered *P. falciparum* DNA PCR detection.

**Results:**

Malaria positive samples were 62/199 (31.2%, 95% CI 24.9, 38.3) by drug seller interpretation of RDT strip, 59/212 (27.8%, 95% CI 22.2, 34.3) by laboratory scientist, 55/212 (25.9%, 95% CI 20.0, 32.6) by RDT nitrocellulose strip PCR and 64/212 (30.2%, 95% CI 24.4, 37.7). The overall agreement between the drug seller interpretation and laboratory scientist re-reading of the RDT strip was 93.0% with kappa value of 0.84 (95% CI 0.75, 0.92). The drug seller compliance with the reported RDT results was 92.5%. The performance of the three diagnostic strategies compared with FTA-PCR as the gold standard had sensitivity between 76.6 and 86.9%, specificity above 90%, positive predictive values ranging from 79.0 to 89.8% and negative predictive values above 90%.

**Conclusion:**

Drug sellers can use RDTs in field conditions and achieve acceptable accuracy for malaria diagnosis, and they comply with the RDT results. *Plasmodium* DNA can be recovered from RDT nitrocellulose strips even in the context of drug shops. Future malaria surveillance and diagnostic quality control studies with RDT cassette as a source of *Plasmodium* DNA are recommended.

**Electronic supplementary material:**

The online version of this article (10.1186/s12936-018-2508-x) contains supplementary material, which is available to authorized users.

## Background

A global increase of 5 million malaria cases in 2016 as compared to the year before [1] calls for improved deployment of available malaria interventions. Up to 445,000 out of an estimated 216 million malaria cases died in 2016 [1]. Prompt diagnosis and case management remains a mainstay of malaria control efforts. It promotes detection of non-malaria illnesses, prevents progression to severe disease or death and halts malaria parasite transmission.

Although, most people who seek treatment for malaria in the government health system receive accurate diagnosis and effective medicines [1, 2], coverage remains low [2]. Only a third (34%) of febrile children are taken to a medical provider in the government-run health sector in sub Saharan countries such as Uganda [1]. The remaining proportion of febrile children seeks care at the community level, from drug sellers and informal health providers.

The global malaria policy of universal testing and treatment aims to detect malaria cases in the first 24 h [3]. Although, blood smear microscopy is the gold standard for malaria diagnosis [4], it is cumbersome, requires experienced technical expertise and consistent availability of consumables and power supply [5]. Thus, it is largely inaccessible for majority of suspected malaria cases in remote rural areas and government health facilities [4, 5]. However, malaria rapid diagnostic tests (RDTs) available as dipsticks or strips can fill this gap [4–8]. They are simple to perform, easily-interpretable and do not require electricity or infrastructural investment [5, 6, 8]. Individuals with no or minimal health training, such as community health workers and some drug sellers can use them [9, 10]. For these reasons, RDTs have been incorporated into the integrated community case management (iCCM) intervention, a WHO/UNICEF recommended community level strategy [10–12] to target antimalarial medicines to parasite confirmed febrile cases. Proper diagnosis of malaria reduces antimalarial drug pressure [4, 7] and triggers the necessity to detect and treat bacterial infections, both of importance in combating antimicrobial resistance [13].

Malaria RDTs are based on immunochromatographic assays with coloured detecting antibodies impregnated on nitrocellulose membranes [5, 6, 8]. They detect specific malaria parasite antigens present in blood of infected or recently infected people [6, 8]. Most widely available RDTs target the histidine-rich protein 2 (HRP2), an antigen that is unique and thus specific to *Plasmodium falciparum* [6, 14]. Other malaria RDTs target parasite-specific lactate dehydrogenase (pLDH) and aldolase [6]. The *Pf*-HRP2 RDTs have better sensitivity and greater thermal stability than pLDH-detecting RDTs [6]. They are suitable for use in malaria endemic areas in sub-Saharan Africa where malaria cases are predominantly due to *P. falciparum* [15].

Correct interpretation of the RDT reading and compliance with the observed result is important to their appropriate use [4, 14]. Studies among drug sellers in Ghana [16], Nigeria (community pharmacies) [17], Uganda [18–23], Tanzania [24], and Myanmar [25] report that RDTs can be safely used, are highly sensitive but have low specificity. For instance, Ansah et al. reports RDT specificity of 30%, 31% and 52% [16], and Mbonye et al., 63.1% [21]. Two studies including one by the authors report a higher positivity rate from RDTs than microscopy, 47% versus 9.3% in South West Uganda [23] and 41% versus 13% in Tanzanian Morogoro region [24], respectively. Mbonye et al. shows one-third of RDT positive cases are malaria negative on expert microscopy and a monthly RDT positivity rate consistently higher than that of microscopy over the 1-year study period [21]. It is not clear whether these observations are due to drug sellers’ conflict of interest for sale of artemisinin-based combination medicines, performance characteristics of the RDT under field conditions or failure of the drug seller to interpret RDT lines. One study reports that community health workers who are traders have six times the odds of prescribing artemisinin-based combination medicines to RDT-negative children than farmers [26]. Also, with the exception of one study in a high malaria transmission setting [21], drug seller interpretation of the RDT reading has not been investigated, especially in the context of low malaria transmission setting. If not addressed, the RDT false positive rates can undermine confidence in the results and diminish the perceived importance of the test and treat guidelines [27].

Additionally, the variation in the observed adherence to RDT results increases the uncertainty about the drug sellers’ ability to interpret RDTs and their performance under field conditions. Although appropriate treatment is moderate (55%) in Nigeria [17] to high (80–99%) in Uganda [19, 21, 23], compliance with negative test results is low. Up to 32% of RDT negative cases in the Ghanaian [16], 37.4% [21] and 45% [20] in the Ugandan, and 51.6% in the Myanmar is studies [25] receive anti-malarial medicines. In the Tanzanian study [24], only 7% of the test negative cases received artemisinin-based combination therapy (ACT).

Therefore, this study was conducted to compare drug seller interpretation of RDTs in the field with the laboratory scientist repeat reading and to assess drug seller compliance with the observed RDT results. The performances of drug seller interpretation and laboratory scientist re-reading of the malaria RDT strips were also compared against polymerase chain reaction (PCR) analysis of *Plasmodium* DNA recovered from dry blood spotted FTA cards. PCR analysis of *Plasmodium* DNA is superior to RDT and is ten times more sensitive [28]. Since *Plasmodium* DNA can be recovered from malaria RDT nitrocellulose strips [28–31], the authors retrieved the RDTs from the study drug shops, extracted DNA from their nitrocellulose strips and analysed it by PCR. The authors argue that this provides an opportunity to incorporate quality assurance into the drug sellers’ use of RDTs.

## Methods

### Participants

The study population consisted of children aged 2–60 months presenting with fever or history of fever, acute respiratory illness and diarrhoeal diseases at two drug shops in Mbarara district. For the purpose of the study each child provided paired blood samples obtained by a finger-prick.

### Setting

Mbarara district is located in South Western Uganda, perceived as a relatively low malaria transmission setting, with malaria parasite prevalence by microscopy among healthy children in the community, averaging between 4.1% [32] and 9.3% [33]. Drug shops in rural areas of Mbarara district were part of a prospective evaluation of the iCCM intervention for paediatric illness in registered drug shops from February 2014 to September 2015. The drug sellers were trained to assess and classify febrile children using clinical signs and symptoms according to the iCCM algorithm. They were given access to anti-malarial drugs (ACT), amoxicillin tablets, zinc sulfate tablets and oral rehydration salts at subsidized prices, RDTs and respiratory count timers free of charge. The iCCM intervention also had a community sensitization component and monthly support supervision by a clinical officer or pharmacist. Details of the iCCM intervention and effects on the health system in Mbarara district have been published elsewhere [23, 34]. Out of a total of 61 drug shops that participated in the iCCM intervention in Mbarara district, two drug shops that recorded most attendance by children less than 5 years were enrolled into the current study, several months after the end of the iCCM intervention, from December 2015 to April 2016.

### Specimen collection

Drug sellers at the study drug shops were trained on how to perform the RDTs according to the revised iCCM intervention guidelines and to collect a blood spot on fast transient analysis (FTA), Whatman™ 3MM filter paper. A child presenting with fever or history of fever was assessed by drug seller to undergo malaria diagnostic testing using the iCCM sick child job aid. A total of 212 duplicate samples of capillary blood were picked from each child, one sample, approximately 5 μL was dispensed into the sample well of the CareStart™ *Pf*-HRP2 RDT cassette (ACCESS BIO, INC. Ethiopian Branch, Yeka, Addis Ababa, Ethiopia), followed by a drop of diluent/buffer into the diluent well, as stated in the manufacturer’s instructions. The drug seller had been instructed to read and interpret the result off the RDT strip after 15–20 min, as stated in the manufacturer’s instructions. The second blood sample, approximately 5 μL, was spotted on a fast transient analysis (FTA), Whatman™ 3MM filter paper in parallel. The blood spotted RDT strips and FTA filter papers were left to dry in open air for up to 30 min, after which they were packaged and stored in transparent zip-lockable polythene bags, labelled with name of under-five (U5) child, drug shop name and date of sample collection.

Drug sellers collected the blood samples as the procedures involved do not require highly trained individuals and equipment such as centrifuge, deep freezer and needles [35, 36]. This technique is advantageous when taking biologic fluid samples from children as it requires small quantities of blood and can use capillary instead of venous blood. Since, dried blood spots on RDT strips and FTA filter paper are stable in tropical climates [36, 37], they were kept at normal working temperatures for up to 2 months before being transferred to the molecular laboratory at the Department of Medical Microbiology, Makerere University College of Health Science. The specimen were protected from adverse conditions of extreme cold or hot [> 40 °C (°Celsius)] temperatures and moisture.

### Nested PCR for detection of *Plasmodium falciparum*

Plasmodium DNA was extracted from blood spot collected on FTA card and RDT strips using Chelex method [30] and QIAamp blood kit (QIAGEN, Inc., Chatsworth, CA), following manufacturer’s instructions. The quantity and quality of the extracted DNA was assessed using Nano Spectrophotometry and QIAxcel advanced automated capillary electrophoresis following the manufacturer’s guideline.

Detection of *P. falciparum* was done using nested PCR that employed two sets of primers that targeted 18S rRNA gene to confirm the genus and species. Nested PCR was performed according to the protocol developed by Snounou et al. in two sequential steps [38]. The first round PCR was performed in 50 µL volume consisting of 1 × of *Taq* 2× Master Mix (New England BioLabs, Massachusetts, USA) of 10 pmol/μL each, of the forward and reverse genus specific outer primers (Table 1), PCR grade water and 5.0 μL of 50 ng/μL template DNA. The reaction was performed with an initial denaturation at 95 °C for 5 min, followed by 35 cycles of 1 min at 94 °C, 2 min at 58 °C, and 2 min at 72 °C, and final extension at 72 °C for 10 min in using a Thermal cycler (Bio-Rad, T100; Singapore).

**Table 1:** Malaria diagnostic strategies used to classify the presence or absence of malaria parasites among the U5 children in the study

No.	Sample/other information	Diagnostic strategy	Remarks
1	Finger prick blood on RDT strip	Drug seller interpretation of RDT strip (drug seller)	Diagnostic test data obtained from drug shop patient registry
2	Retrieved RDT strip at central lab	Laboratory scientist repeat reading of malaria RDT strip (laboratory scientist)	First comparator for drug seller interpretation of the RDT strip
3	*P. falciparum* DNA extracted from the RDT nitrocellulose strip	PCR analysis of *P falciparum* DNA (RDT-PCR)	Used malaria RDT cassettes were transferred from the field to the molecular laboratory at Makerere University College of Health Sciences in Kampala
4	*P. falciparum* DNA extracted from dried blood spot on FTA filter paper	PCR analysis of *P falciparum* DNA (FTA-PCR)	Second reference and gold standard for the other three diagnostic strategies Blood spotted FTA cards were transferred from the field to the molecular laboratory at Makerere University College of Health Sciences in Kampala

Two micro litres (µL) of the first round PCR amplicons were subjected to second PCR using the same amplification conditions and a *P. falciparum*-specific internal primer. The PCR products were analysed using QIAxcel automated capillary electrophoresis. Detection of a 205 bp fragment indicated presence of *P. falciparum*. For each run, DNA from reference *P. falciparum* 3D7 strain and nuclease free water were used as positive and negative controls, respectively. Details of the primers used for nested PCR of 18S rRNA gene in malaria parasites are provided in Additional file 1.

### Costs

The main inputs into the study were RDT cassettes and FTA cards at a cost of $0.5 and $1 per collected sample, respectively. The PCR analysis for *P falciparum* DNA was done at $20 per sample. These costs were met by the research project.

### Variables

The variables included diagnostic test results of samples obtained from U5 children who attended study drug shops during the study period. The blood samples were analysed according to four diagnostics strategies, as shown in Table 1.

Background characteristics of the study population, namely, sex, age, if U5 child presented with diarrhoea and fever or history of fever or any child danger sign were collected. For U5 children presenting with cough, their respiratory rate was recorded. Also data on if U5 child was prescribed oral rehydration salts, zinc-sulfate tablets, dispersible amoxicillin tablets and artemether/lumefantrine tablets by the drug seller was obtained.

### Data analysis

The sample size was estimated using the Buderer formula [39] for research studies designed to evaluate diagnostic tests. This formula takes into account values of sensitivity and the estimated prevalence which were hypothesized to be 95% and 40%, respectively. A 5% level of statistical significance was assumed, which gave a total sample size of 183 research participants. The sample size was further adjusted for 10% incompleteness rate, hence a total of 203 research participants. Blood samples were obtained from a total of 212 U5 children in the current study. Data for 199 children was available from drug shop registries and only 186 observations were complete for the comparison of drug seller interpretation and laboratory re-reading of RDTs.

Data were double-entered into Epidata software version 3.1 (The EpiData Association, Odense, Denmark), cleaned, checked, coded and then transferred to Stata version 13.0 (Stata Corp., College Station, TX, USA) for analysis. First, categorical characteristics of the U5 children enrolled into the study including sex, if U5 child presented with diarrhea and fever or history of fever or any child danger sign, if U5 child was prescribed any child medicines by the drug seller, were summarized as frequencies and proportions. The numerical variable of age of child was summarized by the median and range. The malaria test results observed on all the four diagnostic strategies; namely, drug seller interpretation of RDT strip (Drug seller), laboratory scientist reading of dried and stored RDT strip (Laboratory scientist), PCR analyses of *P. falciparum* DNA extracted from the RDT nitrocellulose strip (RDT-PCR) and FTA card (FTA-PCR), were also presented as frequencies and proportions. Second, drug seller compliance to RDT results was assessed based on the drug seller treatment decision for the febrile or malaria case. Third, the authors calculated sensitivity, specificity, positive predictive values (PPV) and negative predictive values (NPV) [with 95% confidence intervals (CI)] of the three diagnostic strategies; namely, drug seller, laboratory scientist and RDT-PCR strategies compared to the FTA-PCR strategy as the reference method.

Fourth, inter-diagnostic strategy variations for both positive and negative readings were expressed by the percentage of overall agreement and Cohen’s kappa (κ) statistic. A 95% confidence interval (95% CI) was calculated for each κ value using the Stata “KAPPA” module [40].

## Results

### Background characteristics

A total of 212 children aged between 2 and 60 months provided samples for malaria diagnostic testing in the current study. Of these, 103 children presented with cough and were assessed for fast breathing. Data available for 199 U5 children was extracted from the drug shop registries. The median age was 24 months, and slightly more than half (52%) were male. Up to 93% presented with fever or history of fever and 21% presented with diarrhoea (Table 2).

**Table 2:** Background characteristics, medicines prescribed and malaria diagnostic test results among the febrile children attending the study drug shops

Characteristic	Frequency (%)
Sex	
Male	106 (52.7)
Female	95 (47.3)
under-five child symptoms	
Fever	185 (93.0)
Diarrhea	41 (20.6)
Fast breathing^a^	82 (79.6)
Danger sign	4 (2.0)
Medicines prescribed by drug seller to under-five child	
Artemether/Lumefantrine tablets	67 (33.7)
Amoxicillin tablets	84 (42.2)
Oral rehydration salts (ORS)	41 (20.6)
Zinc sulfate tablets	39 (19.6)

^a^Respiratory rate was counted in only U5 children presenting with cough

A total of 212 RDT strips were re-read by laboratory scientist. Of these, 13 RDT strip repeat readings by laboratory scientist were not conclusive. Of the remaining 199 malaria RDT strips, 13 cases did not have corresponding drug seller interpretation record, respectively. Hence, data analysis to compare the drug seller interpretation and laboratory scientist re-reading of RDT strips was done on the 186 cases, excluding a total of 26 child cases (13 child cases that did not have record of the drug seller interpretation and an additional 13 child cases that were not conclusive according to the laboratory scientist re-reading) (Table 3).

**Table 3:** Comparison between drug seller interpretation and the laboratory scientist repeat reading of the RDT strips

Drug seller interpretation of the RDT strip	Laboratory scientist re-reading RDT strip			Methods agreement %, (95% CI)	κ, (95% CI)
	Positive	Negative	Total		
Positive	50	8	58	93.0, (88.3, 96.2)	0.84, (0.75, 0.92)
Negative	5	123	128		
Total	55	131	186		

**Fig. 1:**
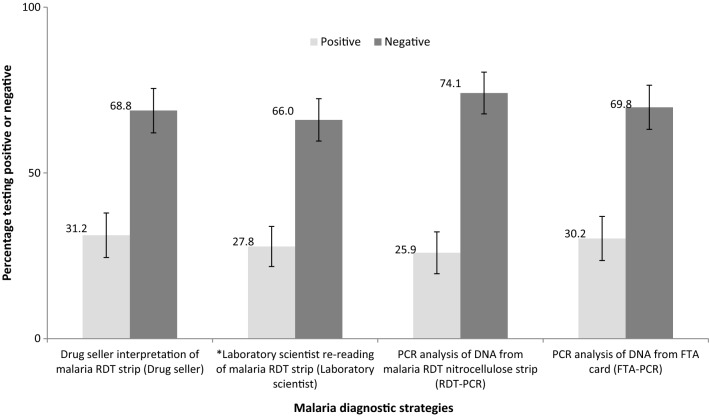
Proportion of malaria cases according to each of the drug seller, laboratory scientist, RDT-PCR and FTA-PCR diagnostic strategies. *Thirteen child cases (6.2%) could not be classified by the laboratory scientist

The proportion of the study population reported positive for malaria parasites were 62/199 (31.2%) by drug seller, 59/212 (27.8%) by laboratory scientist, 55/212 (25.9%) by RDT-PCR and 64/212 (30.2%) by FTA-PCR diagnostic strategies, respectively. For the laboratory scientist diagnostic strategy, 6.1% (13/212) (95% CI 3.6, 10.3) RDT strips could not be classified (see Fig. 1).

### Comparison between drug seller interpretation and laboratory scientist re-reading of the RDT strips

The overall agreement between the drug seller interpretation and laboratory scientist re-reading of the RDT strip was 93% (n = 186) with kappa value of 0.84 (95% CI 0.75, 0.92) (Table 3). The kappa value was 0.81 (95% CI 0.61, 1.00) among malaria positive cases and 0.41 (95% CI 0.11, 0.70) for negative cases, tested by the FTA-PCR diagnostic strategy (the gold standard) (Table 4).

**Table 4:** Comparison between drug seller interpretation and the laboratory scientist repeat reading of the RDT strips, stratified by the FTA-PCR (gold standard) test result

FTA-PCR test result	Drug seller strategy proportion	Laboratory scientist strategy proportion	Methods agreement (%), (95% CI)	κ, (95% CI)
Positive on FTA-PCR	46/57	49/57	94.7 (85.4, 98.9)	0.81 (0.61, 1.00)
Negative on FTA-PCR	117/129	123/129	92.3 (86.2, 96.2)	0.41 (0.11, 0.70)

Of the five cases reported negative by drug seller but positive by laboratory scientist, three tested positive and two negative by FTA-PCR (the gold standard), respectively. Only one out of these five cases was prescribed ACT medicines by drug seller. All the eight cases reported positive by drug seller but negative by laboratory scientist, tested negative by FTA-PCR. Four of these eight cases were prescribed ACT medicines by the drug seller.

Additionally, Table 5 presents the comparison of the two diagnostic strategies of drug seller and laboratory scientist, including the 13 RDT strips that were inconclusive according to the laboratory scientist diagnostic strategy. The agreement between the drug seller and laboratory scientist diagnostic strategy decreased from 93% (95% CI, 88.3, 96.2) to 86.9% (95% CI, 81.4, 91.3). The kappa value decreased from 0.84 (95% CI, 0.75, 0.92) to 0.72 (95% CI, 0.61, 0.83).

**Table 5:** Comparison between drug seller interpretation and the laboratory scientist repeat reading of the RDT strips, including cases that could not be classified by laboratory scientist

Drug seller interpretation of the RDT strip	Laboratory scientist re-reading malaria RDT strip				Methods agreement %, (95% CI)	κ, (95% CI)
	Positive	Negative	Not conclusive	Total		
Positive	50	8	4	62	86.9, (81.4, 91.3)	0.72, (0.61, 0.83)
Negative	5	123	9	137		
Total	55	131	13	199		

Among the 13 child cases that were inconclusive by the laboratory scientist diagnostic strategy, 9 tested malaria positive and 4 were malaria negative by the drug seller diagnostic strategy. However, when tested by the FTA-PCR diagnostic strategy, 10 child cases tested negative while 3 cases tested positive for malaria parasites.

### Drug seller compliance with the reported RDT results

The drug seller compliance with the reported RDT results was 92.5% (Table 6). Compliance among malaria positive cases was 91.9% while that among negative cases was 92.7%, respectively. Up to 8.1% malaria positive cases did not receive ACT medicines, while 7.3% negative cases received ACT medicines.

**Table 6:** Drug seller compliance with the reported RDT results

Drug seller interpretation of the RDT strip	Drug seller prescribed ACT medicines for the child			Compliance (%), (95% CI)
	Yes	No	Total	
Positive	57	5	62	92.5, (87.9, 95.7)
Negative	10	127	137	
Total	67	132	199	

### Comparison of the diagnostic strategies against PCR analysis for detection of *P. falciparum* DNA extracted from FTA card

The sensitivity of the drug seller, laboratory scientist and RDT-PCR diagnostic strategies when compared to FTA-PCR strategy for *P. falciparum* detection varied from 77% (95% CI 64.3, 86.2) for RDT-PCR to 87% (95% CI 75.8, 94.2) for the laboratory scientist strategy. The specificity of the three diagnostic strategies was > 90% when compared to the gold standard (FTA-PCR). The positive predictive values were 89% for both RDT-PCR and laboratory scientist and 79% for the drug seller strategy. The negative predictive values were > 90% with the highest being 94.3% (95% CI 89.1, 97.5) for the laboratory scientist strategy. The three diagnostic strategies of drug seller, laboratory scientist and RDT-PCR showed high measure of overall agreement > 87% with the gold standard and κ value (kappa statistic) between 0.70 and 0.85 (Table 7). Details of the formulae and numbers used to calculate the sensitivity, specificity, predictive values and likelihood ratios of the diagnostic strategies are provided as supplementary material (Additional file 2). The kappa statistic for the average agreement over all pairs of the diagnostic strategies was 0.74 (95% CI 0.71, 0.76).

**Table 7:** Comparison of performance of drug seller, laboratory scientist and RDT-PCR diagnostic strategies against PCR detection of *P. falciparum* DNA extracted from FTA card (FTA-PCR)

Diagnostic strategy	Sensitivity %, (95% CI)	Specificity %, (95% CI)	Predictive value		Likelihood ratio		Methods agreement %, (95% CI)	κ, (95% CI)
			Positive test %, (95% CI)	Negative test %, (95% CI)	Positive test %	Negative test %		
Drug seller interpretation of RDT strip (drug seller)	81.7, (69.6, 90.5)	90.6, (84.5, 94.9)	79.0, (66.8, 88.3)	92.0, (86.1, 95.9)	8.7	0.2	87.9, (82.6, 92.1)	0.72, (0.61, 0.82)
Laboratory scientist re-reading of RDT strip (laboratory scientist)	86.9, (75.8, 94.2)	95.7, (90.8, 98.4)	89.8, (79.2, 96.2)	94.3, (89.1, 97.5)	20.0	0.1	92.9, (88.5, 96.1)	0.83, (0.75, 0.92)
PCR analysis of *P. falciparum* DNA extracted from RDT nitrocellulose strip (RDT-PCR)	76.6, (64.3, 86.2)	95.9, (91.4, 98.5)	89.1, (77.8, 95.9)	90.4, (84.7, 94.6)	18.9	0.2	90.1, (85.3, 93.8)	0.76, (0.66, 0.85)

## Discussion

The findings from this study demonstrate that drug seller interpretation has high concordance with laboratory scientist re-reading of the malaria RDT strips. This finding is similar to that of another Ugandan study [21] that reports 95% agreement between drug seller and research team reading of RDT strips, despite a higher malaria transmission setting than that of the current study. Furthermore, the laboratory scientist diagnostic strategy used as a reference in the current study performs acceptably well against a proven more sensitive diagnostic method—the PCR analysis of *P. falciparum* DNA extracted from FTA card (FTA-PCR). The high agreement between drug seller and the laboratory scientist strategies in the current study is probably a result of the drug sellers having undergone training in the iCCM intervention study [23]. The iCCM intervention involved hands-on training of the drug sellers on the use of RDT strips to detect presence of malaria parasites, monthly support supervision by a clinical officer or pharmacist and an enhanced diagnostics and medicines supply mechanism [23]. Other studies among community health workers acknowledge that successive practice improves RDT use [41]. Given the relatively low malaria transmission setting, the authors of the current study had hypothesized a higher rate of false positives by drug sellers so as to rationalize their sale of anti-malarials.

The current study also demonstrates high compliance to the RDT results by the drug sellers. This is probably due to the implementation of co-interventions in the drug shops prior to the current study. In addition to drug seller training and support supervision, enhanced supply mechanism and subsidies, communities were sensitized on importance of accepting the RDT test outcomes, as part of the iCCM intervention conducted from February 2014 to September 2015 [23, 34]. The high compliance observed is possibly a residual effect. These findings are consistent with those from similar studies conducted among drug sellers [21, 23], especially for RDT-positive cases [16, 20, 22, 24]. Also studies among community health workers in Tanzania [42], Malawi, Senegal [43] report similarly high compliance levels to RDT results. However, some drug seller studies report variable inappropriate treatment of RDT-negative cases with anti-malarial medicines: 45% [20], 10% [22], 1.5% [21] in each Ugandan study, 7% in Tanzania [24] and 3% in Ghana [16] of the RDT-negative cases are given anti-malarials, respectively. In community health worker studies, greater than 10% of the RDT negative results [10, 11, 44, 45] and up to 20% [46], 30% [47] and 58% [48] were prescribed ACT. Clinical judgement by community health worker is advanced to justify giving ACT to test negative cases [47].

Compliance with observed malaria RDTs results in the current study is also higher than 83% (95% CI 80, 86) reported by Kabaghe et al. in a systematic review that analysed pooled findings from 14 studies involving clinicians and community health workers [49]. In that study, compliance among the malaria positive cases is higher 97% (95% CI 94, 99) while that among negative cases is lower 78% (95% CI 66, 89) [49] than reported in the current study, respectively. The difference in these findings is probably a result of less trained health cadres being more trusting in RDT results while clinicians rely more on symptoms and past experience [50]. For instance, Kabaghe et al. also reports a higher compliance, 95% (95% CI 92, 98) among RDT negative cases among community health workers than 75% (95% 58, 89) among clinicians [49].

In the context of the current study, the drug seller interpretation of the RDTs and subsequent prescription or non-prescription of ACT, does not appear to have been substantially influenced by the drug seller motive to maximize sales. As chronicled in a related publication [34], their primary interest for using the RDTs was the improved reputation within the community as a result of the increased trust in the healthcare provided to the U5 children.

The drug seller diagnostic strategy showed a sensitivity of 82% and specificity of 91% when using the FTA-PCR strategy as a gold standard. PCR is an ideal gold standard as it is based on identification of *Plasmodium* genus and species specific markers [51]. It has capability to detect malaria parasites at densities as low as 0.002 parasites/µL [52, 53] as compared to RDTs whose detection level varies from 100 to 200 parasites/µL [54]. The lower sensitivity of the drug seller diagnostic strategy observed in the current study could be due to low parasitaemia in some of the study participants. However, individuals with low parasitaemia remain a public health threat as they can contribute to *P. falciparum* transmission dynamics [52, 53]. Similarly, the sensitivity and specificity of laboratory scientist re-reading of RDT strips though higher than those of drug seller interpretation were 87% and 96%, respectively, when compared to FTA-PCR as a gold standard. The better performance of the laboratory scientist is likely due to differences in health provider characteristics. For instance, subjective interpretation by [55] and poor eye sight of [45] the health provider can lead to poor performance.

In the current study, the observed sensitivity of the laboratory scientist strategy is higher than estimates from similar studies [56, 57]. An evaluation of *Pf*-HRP2 RDT (CareStart™) using samples from suspected malaria cases aged 1–81 years using PCR as the reference, reports sensitivities ranging from 71% [56], 72% to 75% [57] as compared to 87% in the current study. The difference in findings could be explained by the inclusion of study participants of all ages yet the current study analysed samples taken from children 5 years old or less. Getnet et al. reports that sensitivity of RDTs when compared to PCR as reference is higher in study population under 18 years than older ages [56]. On the contrary, an evaluation of *Pf*-HRP2 malaria RDTs (MAKROmed™) against PCR as reference among 853 women in Burkina Faso reports almost similar sensitivity of 90% [58] as the current study. The specificity of the laboratory scientist re-reading of the RDT strips against PCR as reference in the current study is the same as observed in north-west Ethiopia (95%) [56], but higher than estimates from western Kenya (82%) [57] and Burkina Faso (65%) [58].

The variability in sensitivity and specificity of *Pf*-HRP2 RDTs as observed in the current study and other studies [56–59] warrants a larger scale examination of the magnitude of the misclassification with current RDTs. Moreover, these field estimates are below the minimum WHO thresholds for sensitivity and specificity of 95 and 90% for all malaria species, respectively [60]. Possible explanatory factors for this variation include genetic variation in the histidine rich proteins, batch quality variations [54], *Pf*-HRP2 persistence in blood following parasite clearance [61], and *Pf*-HRP2 gene deletions [15, 62, 63]. Other predictors are parasite density, environmental and several host factors, inter-lot variability and performance deterioration, that could result from storage or transportation conditions [6, 8, 54]. Therefore, an external quality assurance scheme for RDT use under field conditions, to detect any failures early and minimize adverse effects of misdiagnosis of malaria is urgently needed. RDT cassettes in routine practice can form the basis of such a scheme as they are an excellent source of *Plasmodium* DNA especially in the context of drug shops [29–31].

The findings show high PPVs of 79% for the drug seller strategy, and 89% each for the laboratory scientist and RDT-PCR diagnostic strategies. The observed PPVs in the current study are consistent with findings from a Tanzanian study in which RDTs of samples from febrile children 2–59 months were compared with PCR as a [64]. However, the PPVs for laboratory scientist and RDT-PCR strategies are higher than 82% that is reported in Wanja et al., except for the PPV of the drug seller strategy which is the same [57]. The NPVs for drug seller, laboratory scientist and RDT-PCR diagnostic strategies in the current study are high (> 90), and above 73–76% that is reported by a Kenyan study [57], but lower than 98.5% that was observed in the Tanzanian study [64]. The difference between predictive values in the current study and other similar studies could be due to differences in the underlying malaria prevalence [65], which in turn is affected by seasonal variations. Given that the prevalence of malaria parasites in the current study population is between 25 and 31% (Fig. 1), the observed predictive values are high and indicative of the probability of disease given the observed results of the test for each of the diagnostic strategies.

The RDT-PCR diagnostic strategy showed low sensitivity of 77% and high specificity of 96% as compared to FTA-PCR strategy. This lower sensitivity of the RDT-PCR could be a result of loss of parasite DNA when the blood sample is lysed and diluted before migration on the nitrocellulose membrane in the RDT cassette [28]. Additional loss could be due to the dispersion on the blotting paper [28]. The amount of DNA recovered is also dependent on the region of the nitrocellulose strip considered for the extraction reaction. Studies show that DNA is concentrated in the region approximately half way between the blood application site and the result lines [66]. Regardless, findings from the current study support the use of RDT cassettes as a source of parasite DNA in place of dry blood spots on FTA cards/filter paper, for an external quality assurance scheme of RDT use in drug shops. Routine collection and PCR analysis of dried RDTs can thus be introduced as part of quality control. In comparison to recovery of *Plasmodium* DNA from blood smears where contamination can occur during staining, microscopic examination or storage, the risk of contaminating RDT cassettes is minimal [67].

This study has notable limitations. First, the current study compared the RDT based on detection of *P. falciparum*-specific HRP2 antigen in blood with PCR detection of *Plasmodium* DNA extracted from dried blood spots. The two diagnostic strategies have inherent differences in their mechanism of detection. Additionally, sensitivity of the *Pf*-HRP2 RDT decreases at parasite densities below 500 parasites per microlitre of blood [5, 68], while PCR analysis remains sensitive at parasite densities as low as 10 parasites per microliter of blood [69]. Despite this, the authors observed substantial agreement between PCR analysis of *Plasmodium* DNA from FTA card and drug seller interpretation of RDT strip of 72%, and laboratory scientist re-reading of 83%. The difference in agreement could have been a result of the discrepancy in the mechanism of parasite detection explained above. However, a higher agreement between our initial comparator (laboratory scientist re-reading) and the more sensitive PCR analysis of parasite DNA lends credence to the authors’ approach to evaluating performance of RDT by drug sellers in field conditions.

Second, the nature and context of the encounters between the U5 children who provided blood samples and the drug sellers in the current study is not representative of the typical drug shop setting. The drug sellers in the current study were trained on how to use the malaria RDT, the iCCM treatment algorithm and were monitored and supervised on a monthly basis for 20 months before the evaluation of these malaria diagnostic strategies [23]. This implies that the competency of drug sellers in the current study to interpret and comply with RDT results is probably higher [41] than what would be observed among the typical drug sellers described in other studies [70, 71]. Potentially, drug sellers in the current study were not prone to errors in testing procedures which could have led to poor performance of RDTs [18]. Lastly, health providers in private sector in poor rural settings are heterogeneous, context-specific and operate in constantly changing environments [34].

Third, the current study did not perform malaria microscopy and hence parasite density is not reported. The authors argue that the study setting is homogenous with a relatively low malaria transmission. Hence, no observations can be made about the performance of RDTs at different parasite densities or malaria transmission levels. Other studies have shown that the sensitivity of the RDTs lowers with a decrease in parasite densities [64] and the predictive values for a positive test are affected by the prevalence of the disease [65]. The current study excluded *Plasmodium* species differentiation and evaluated only the performance of malaria diagnostic strategies in detecting *P. falciparum* malaria. Since 99% of the malaria infection in Uganda is due to *P. falciparum* [1, 72], the need for species differentiation was minimal.

## Conclusion

Malaria RDTs in the hands of drug sellers in field conditions diagnose malaria among U5 children with acceptable accuracy. Additionally, drug sellers comply with the RDT results, especially when supported with other co-interventions as present in the iCCM intervention. However, the good drug seller performance in malaria diagnosis and compliance to RDT results can be undermined by the high false positivity rate due to low specificity implicit to the *Pf*-HRP2 RDT platform or caused by drug seller related factors. Further research to test an external quality assurance scheme for RDT use under field conditions based on nucleic acid amplification of *Plasmodium* DNA recovered from the RDT nitrocellulose strip is, therefore, warranted.

## Additional files

**Additional file 1.** Primers for nested PCR of 18S rRNA gene in malaria parasites.

**Additional file 2.** The calculations of sensitivity, specificity, predictive values and likelihood ratios of the diagnostic strategies and respective formulae used.

## Abbreviations

ACT: artemisinin-based combination therapy
CHWs: community health workers
DNA: deoxyribonucleic acid
FTA: fast transient analysis
HRP2: histidine rich protein 2 antigen
IEC: information, education and communication
iCCM: integrated community case management intervention
MoH: Uganda Ministry of Health
NMCP: National Malaria Control Programme
NPV: negative predictive value
ORS: oral rehydration salts
PCR: polymerase chain reaction
pLDH: parasite-specific lactate dehydrogenase
PPV: positive predictive value
RDT: rapid diagnostic test
SDG: Sustainable Development Goals
U5: under-five
UNICEF: United Nations Children’s Fund
VHTs: Village Health Teams
WHO: World Health Organization
^o^C: degrees Celsius
