# Supplementary material for: Can malaria rapid diagnostic tests by drug sellers under field conditions classify children 5 years old or less with or without Plasmodium falciparum malaria? Comparison with nested PCR analysis
Source: Malar J. 2018 Oct 16;17:365. doi: 10.1186/s12936-018-2508-x (PMC6192306; doi:10.1186/s12936-018-2508-x)
Supplement: Supplementary file 1 — Additional file 1. Primers for nested PCR of 18S rRNA gene in malaria parasites. [file 12936_2018_2508_MOESM1_ESM.docx]

Additional file 1: Table S1. Primers for nested PCR of 18S rRNA gene in malaria parasites

| **Types of PCR** | **Primer name** | **Sequence (5’–3’)** | **Species** |
| --- | --- | --- | --- |
| Nested – first | rPLU6 | TTAAAATTGTTGCAGTTAAAACG | *Plasmodium sp.* |
|  | rPLU5 | CCTGTTGTTGCCTTAAAC TTC |  |
| Nested – second | rFAL1 | TTAAACTGGTTTGGGAAAACCAAATATATT | *P. falciparum* |
|  | rFAL2 | ACACAATGAACTCAATCATGACTACCCGTC |  |
